# Is negative density‐dependent reproduction regulated by density‐induced stress in root voles? Two field experiments

**DOI:** 10.1002/ece3.8927

**Published:** 2022-05-13

**Authors:** Guozhen Shang, Shouyang Du, Yanbin Yang, Yan Wu, Yifan Cao, Jianghui Bian

**Affiliations:** ^1^ Key Laboratory of Adaptation and Evolution of Plateau Biota Northwest Institute of Plateau Biology Chinese Academy of Sciences Xining China; ^2^ Qinghai Key Laboratory of Animal Ecological Genomics Xining China; ^3^ College of Animal Science and Veterinary Medicine Henan Institute of Science and Technology Xinxiang China; ^4^ College of Veterinary Medicine Henan Agricultural University Zhengzhou China; ^5^ School of Life and Environment Sciences Hangzhou Normal University Hangzhou China

**Keywords:** density, FCM level, negative density‐dependent reproduction, reproduction, root vole, stress

## Abstract

Density dependence in reproduction plays an important role in stabilizing population dynamics via immediate negative feedback from population density to reproductive output. Although previous studies have shown that negative density‐dependent reproduction is associated with strong spacing behavior and social interaction between individuals, the proximal mechanism for generating negative density‐dependent reproduction remains unclear. In this study, we investigated the effects of density‐induced stress on reproduction in root voles. Enclosed founder populations were established by introducing 6 (low density) and 30 (high density) adults per sex into per enclosure (four enclosures per density in total) during the breeding season from April to July 2012 and from May to August 2015. Fecal corticosterone metabolite (FCM) levels, reproductive traits (recruitment rate and the proportion of reproductively active individuals), and founder population numbers were measured following repeated live trapping in both years. The number of founders was negatively associated with recruitment rates and the proportion of reproductively active individuals, displaying a negative density‐dependent reproduction. FCM level was positively associated with the number of founders. The number of founder females directly affected the proportion of reproductive females, and directly and indirectly through their FCM levels affected the recruitment rate; the effect of the number of male founders on the proportion of reproductive males was mediated by their FCM level. Our results showed that density‐induced stress negatively affected reproductive traits and that density‐induced stress is one ecological factor generating negative density‐dependent reproduction.

## Graphical abstract

Negative density‐dependent reproduction plays an important role in stabilizing population dynamics via immediate negative feedback from population density to reproductive output. In this study, we tested the hypothesis that negative density‐dependent reproduction in voles may be due to the suppressive effects of density‐induced stress on reproduction. We conclude that density‐induced stress is one ecological factor generating negative density‐dependent reproduction effects in vole populations.

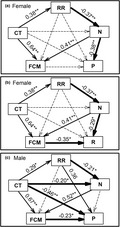

## 1 INTRODUCTION

Negative density‐dependent reproduction refers to the negative effects of population density on fecundity during breeding season; that is, the higher the density, the lower the fecundity (Arcese & Smith, 1988; Both, 1998; Coulson et al., 2000; Dhondt et al., 1992; Focardi et al., 2002; Møller, 1989; Wauters & Lens, 1995). It is a very common observation in seasonal territorial microtine rodents, such as the grey‐sided vole (*Clethrionomys rufocanus*) (Saitoh, 1981), meadow vole (*Microtus pennsylvanicus*) (Ostfeld et al., 1993), water vole (*Arvicola terrestris*) (Saucy, 1994), common vole (*Microtus arvalis*) (Inchausti et al., 2009), bank vole (*Clethrionomys glareolus*) (Koskela et al., 1999), and Yangtze vole (*M*. *fortis calamorum*) (Zhang et al., 2010), and plays an important role in stabilizing population dynamics via immediate negative feedback from population density to reproductive output (Ostfeld et al., 1993). The mechanisms underlying negative density‐dependent reproduction are associated with direct and indirect interactions among individuals, such as consumptive (i.e., resource‐based) and non‐consumptive (i.e., social‐based) competition (Edeline et al., 2010; Mugabo et al., 2017; Rödel et al., 2004; Saitoh et al., 1997), as well as social suppression of juvenile maturation (Saitoh, 1981).

Christian (1971) suggested that negative density‐dependent reproduction might be the result of increased adrenocorticotropic hormone secretion in response to increased density, which then decreased or suppressed reproduction. However, Christian's research has been doubted because it was largely performed on captive animals in a laboratory setting (Krebs & Myers, 1974; Lee & McDonald, 1985); moreover, there has been limited evidence supporting their hypothesis. Nonetheless, studies have shown that factors known to influence negative density‐dependent reproduction, such as social interference and antagonistic interactions, are normally modulators of individual stress responses in the population and act as stressors to activate the hypothalamic–pituitary–adrenal axis and subsequent secretion of glucocorticoids (GC), a critical hormone in stress response (Sheriff et al., 2009). Studies on free‐living, non‐social, territorial vertebrates demonstrated that population density is usually positively associated with GC levels (see review by Creel et al., 2013, but see Charbonnel et al., 2008; Harper & Austad, 2004; Kuznetsov et al., 2004). In voles especially, increase in population density was associated with an increase in corticosterone levels (Bian et al., 2015; Boonstra & Boag, 1992; Novikov & Moshkin, 1998). For some social mammals living in groups, dominant individuals have higher reproductive success and heightened GCs level (see review by Sapolsky, 2005); however, evidence from laboratory studies and free‐ranging iteroparous territorial rodents have shown that chronically elevated GC concentrations inhibit the gonadal axis function and lead to a negative effect on reproduction (see review by Creel et al., 2013). Thus, negative density‐dependent reproduction in microtine rodents may be the result of elevated corticosterone concentrations in response to increased density.

In the present study, we investigated the effects of density‐induced stress on reproduction via manipulation of the population density in the root vole (*Microtus oeconomus*). This is part of a larger multifaceted project examining the effects of density‐induced maternal stress on population dynamics. Parental populations for breeding offspring were established by introducing 6 and 30 adults of each sex in low‐ and high‐density enclosures, respectively, in 2012 and 2015. Our previous papers have reported that the population under high‐density conditions showed higher mean fecal corticosterone metabolite (FCM) levels than the population under low‐density conditions in both 2012 (Bian et al., 2015) and 2015 (Yang et al., 2018). In this study, we present unpublished data from the same parental populations in 2012 and 2015, which include recruitment rate and proportion of reproductively active individuals and the numbers of male and female founders in both years. We separately tested the difference in reproductive traits between high‐ and low‐density parental populations, and used the recursive model in the structural equation model (SEM) to analyze how the number of founders and FCM levels affected reproduction. We aimed to test the hypothesis that negative density‐dependent reproduction in voles may be due to the suppressive effects of density‐induced stress on reproduction. We predicted that the high‐density population would have a lower recruitment rate and a lower proportion of reproductively active individuals, and the effects of founder number on both reproductive traits would be mediated by FCM levels.

## 2 MATERIALS AND METHODS

### 2.1 Root voles in the study area

Our study was conducted at Haibei Alpine Meadow Ecosystem Research Station, Menyuan County, approximately 155 km north of Xining, the capital city of Qinghai province, People's Republic of China (37°370'N, 101°120'E). The area is a secondary vegetation‐type meadow with a dense leaf layer. The major plant species include *Elymus nutans*, *Poa* sp., *Kobresia humilis*, and *Potentila fruticosa*. The root vole is the most common rodent in the study area. Root vole populations in this area fluctuate only seasonally, with the lowest levels occurring in early spring; multiyear cycles are weak or absent (Jiang et al., 1991). Root voles have a preference for dense vegetation (mainly *E*. *nutan*s) (Bian et al., 1994; Liu et al., 1991). The average population size across the study sites ranged from 70 to 170 voles ha^−1^ during the past 20 years, while in certain dense grassland sites, where grazing activities were limited and vegetation consisted mainly of *E*. *nutans*, the density reached c. 400 voles ha^−1^ in late autumn (high level season, Bian et al., 1994; Jiang et al., 1991; Sun et al., 2002). The breeding season typically lasts from April to late October. Females have exclusive territoriality during the breeding season; males, conversely, have large area ranges that extensively overlap with those of other males (Sun et al., 1982). The lifetime of free‐ranging individuals is <1 year. Spring‐born individuals attain sexual maturity in the year they are born; fall‐born voles remain reproductively inactive during winter (Bian et al., 2015).

### 2.2 Experimental facility

The experiment was undertaken in eight 0.15‐ha (50 × 30 m) outdoor enclosures in 2012 and 2015. The enclosures were constructed using galvanized steel panels (1.5 m aboveground and 0.5 m belowground), which prevented mammalian predators from gaining entry. Avian predators were excluded by a 3 × 3 cm grid wire mesh held aloft by a central pillar (10 × 250 cm) in each enclosure. Each enclosure was equipped with 60 laboratory‐made wooden traps (Bian et al., 2015), spaced in a 5 × 5 m grid. Each trap was covered with a wooden sheet to protect it from exposure to precipitation and temperature extremes.

### 2.3 Establishment of populations and live trapping

A total of 288 voles of each sex, 6 months of age or older, were separately used to establish the enclosure populations in 2012 and 2015. They were either F2 generations born in the laboratory or captured as juveniles in the previous year. All individuals were tagged in the ear with identifying metal tags. The populations were introduced into the enclosures in April 2012 and May 2015 at two density conditions. According to the low‐ and high‐density levels observed in nature (Bian et al., 1994; Jiang et al.,^1991^; Sun et al., 2002), the low‐density condition consisted of 6 adults per sex in each of the four enclosures, and the high‐density condition consisted of 30 adults per sex in each of the other four enclosures in 2012 and 2015. The initial body weights did not differ among the voles in the different enclosures (*F*_7, 280_ = 1.72, *p* = .103 in 2012, *F*_7, 280_ = 0.192, and *p* = .987 in 2015). Live trapping started after allowing the animals to acclimate to their new environments for 2 weeks and lasted until late July 2012 and August 2015, respectively. Standard capture–mark–recapture methods were used throughout the study. Six trapping sessions were conducted in 2012 and seven in 2015; each consisted of three trapping days. The time interval between any two trapping sessions was 1 week. The traps were set between 7:00 a.m. and 7:00 p.m., baited with a bit of carrot, checked every 2 h, and locked when trapping did not occur. Following each capture, we recorded animal identification, sex, and body mass. Females were considered reproductive if they had enlarged nipples and teats barren of hair. Males were considered in breeding conditions if their testes were scrotal rather than abdominal. The animal was, then, released at the point of capture after handling. The F1 offspring born in the enclosures were captured at 20–30 days of age and permanently moved to the laboratory for use in subsequent experiments (Bian et al., 2015; Yang et al., 2018).

### 2.4 Fecal corticosterone metabolite measurement

Fecal corticosterone metabolite levels reflect the level of circulating corticosterone that occurred 10–12 h previously in root voles (He et al., 2013), and FCM is derived primarily from plasma‐free corticosterone in rodents (Sheriff et al., 2010). Fecal samples for the FCM analysis were collected during the first 2 h of trapping (09:00–11:00 a.m.), and each captured animal was sampled once within a 3‐day trapping session; thus, all animals provided only a single sample in each trapping session. Meanwhile, each trap was cleaned with water before collecting the fecal sample, ensuring that the samples were not influenced by the previous trapping or time of day. Traps used to sample feces only had a few carrots. Fecal samples from pregnant females were not collected to avoid confounding effects of reproduction states on FCM levels (Edwards et al., 2019; McDonald, 1998). The total number of fecal samples was 546 and 832 in 2012 and 2015, respectively, throughout each experiment, and they accounted for 59% and 67% of the sum of minimum number known alive in each trapping session throughout the duration of experiments in both years (excluding reproductive females). The collected samples were, then, frozen in ice, transported to the laboratory, and stored in a −20°C freezer until analysis. FCM was measured following the methods outlined by Yang et al. (2018), previously validated for root voles. First, the collected fecal samples were lyophilized (Labconco, Kansas City, MO, USA) for 14–18 h, ground into particles, and homogenized in 0.5 ml NaOH solution (0.04 M). The extraction of FCM was performed by adding 5 ml of CH_2_Cl_2_ to the sample (0.1 g), followed by sonication for 15 min (Pihl & Hau, 2003), and centrifugation for 15 min at 3000 *g*. After centrifugation, 1 ml of the solution was taken from the organic layer, diluted with 3 ml CH_2_Cl_2_, and then mixed with 4 ml of a mixed solution of sulfuric acid and ethanol (7:3, v:v). The samples were, then, shaken for 2 min and rested for 30 min before separation of the sulfuric acid layer for fluorescence detection. The fluorescence density in each sample was measured using an RF‐540 IPC Fluorometer (Shimadzu, Japan) at excitation and emission wavelengths of 470 and 520 nm, respectively, and the FCM concentration in each sample was calculated based on the fluorescence densities produced by varying concentrations of the standard (Chen et al., 2012).

### 2.5 Statistical analysis

We used the minimum number known alive (MNKA) method to estimate the founder numbers. The recruitment rate was calculated as the recruits captured in a trapping session divided by the adult females captured in the second preceding session in each enclosure. The proportion of the reproductively active individuals was evaluated using the numbers of reproductively active voles divided by the total numbers of adults captured for each sex in a trapping session. Recapture rate was calculated as the numbers of captured individual divided by MNKA in a trapping session.

We used generalized linear mixed models (GLMMs) in SPSS v.19 (IBM, Armonk, NY, USA) to test the effects of population density on founder number, reproduction, and recapture rate. We combined both years’ data to increase the statistical power. Founder number, proportion of reproductively active individual, recruitment rate, and recapture rate as response variables; the treatment, sex, and time as predictor variables for founder numbers analyses and treatment; and time as predictor variables for other data analyses, predictor variables were entered in all the models to test separately the main and interactive effects. In all data analyses, fence and year were both specified as random effects, which allowed for correlated responses within years and fences. Factor fence was nested within factor year. Because founder number is subject to Poisson distribution, response variables were analyzed using Poisson distribution and log‐link function. Because the number, recruitment rate, proportion of reproductively active individuals, and recapture rate were repeatedly sampled during the experiment, these data were analyzed using GLMM repeated measures to take account of the change in variables over time. For this method, because response variables may be correlated with observational units (enclosures) at different time points (trapping session), we first conducted a comparison of candidate models with various covariance structures using the corrected Akaike information criterion (AICc). The model with the smallest AICc value was then selected. Post hoc comparisons for significant treatment effects were followed by the Bonferroni test. Comparisons of the means were considered significant at *p* < .05. All data are expressed as mean ± standard error.

Recursive model in SEM is a structural equation model that described the complex relationship between variables by simultaneous equations; all the paths flow one way with no feedback or reciprocal loops and the errors are uncorrelated (Schreiber, 2008). The variables in recursive model include endogenous variables (outcome variables) and exogenous variables (predictor variables). Different from regression analysis, the recursive model can explain the direct and indirect effects between variables, and the path graph can more intuitively represent the complex relationship between variables. To tease apart which factors of density and FCM levels induced reproductive suppression, we used the model to explore the pathways of how density, through FCM level of founder voles, affected reproductive traits (recruitment rate and proportion of reproductively active individuals). We first considered a full model that included all possible pathways, and, then, sequentially eliminated non‐significant pathways until we attained the final model. We reported path coefficients as standardized effect sizes. This analysis was performed with a longitudinal data set, which included cumulative time of trapping session (CT), recapture rate (RR), founder number (N), recruitment rate (R), proportion of reproductively active individuals (P), and mean FCM level per trapping session in 2012 and 2015, respectively. In analyses, RR, N, FCM, R, and P were entered in the model as endogenous variables, and CT as exogenous variables. Because founder number, recapture rate, and proportion of reproductively active individuals are not subject to normal distribution, the data were sqrt transformed and arcsine transformed prior to the analyses, respectively. We used the *χ* ^2^ test (if *p* > .05, then no paths were missing, and the model was a good fit) and root mean square error of approximation (RMSEA) (if *p* < .05, then no paths were missing, and the model was a very good fit) to evaluate the fit of the model.

## 3 RESULTS

### 3.1 Density and recruitment rate

**FIGURE 1:**
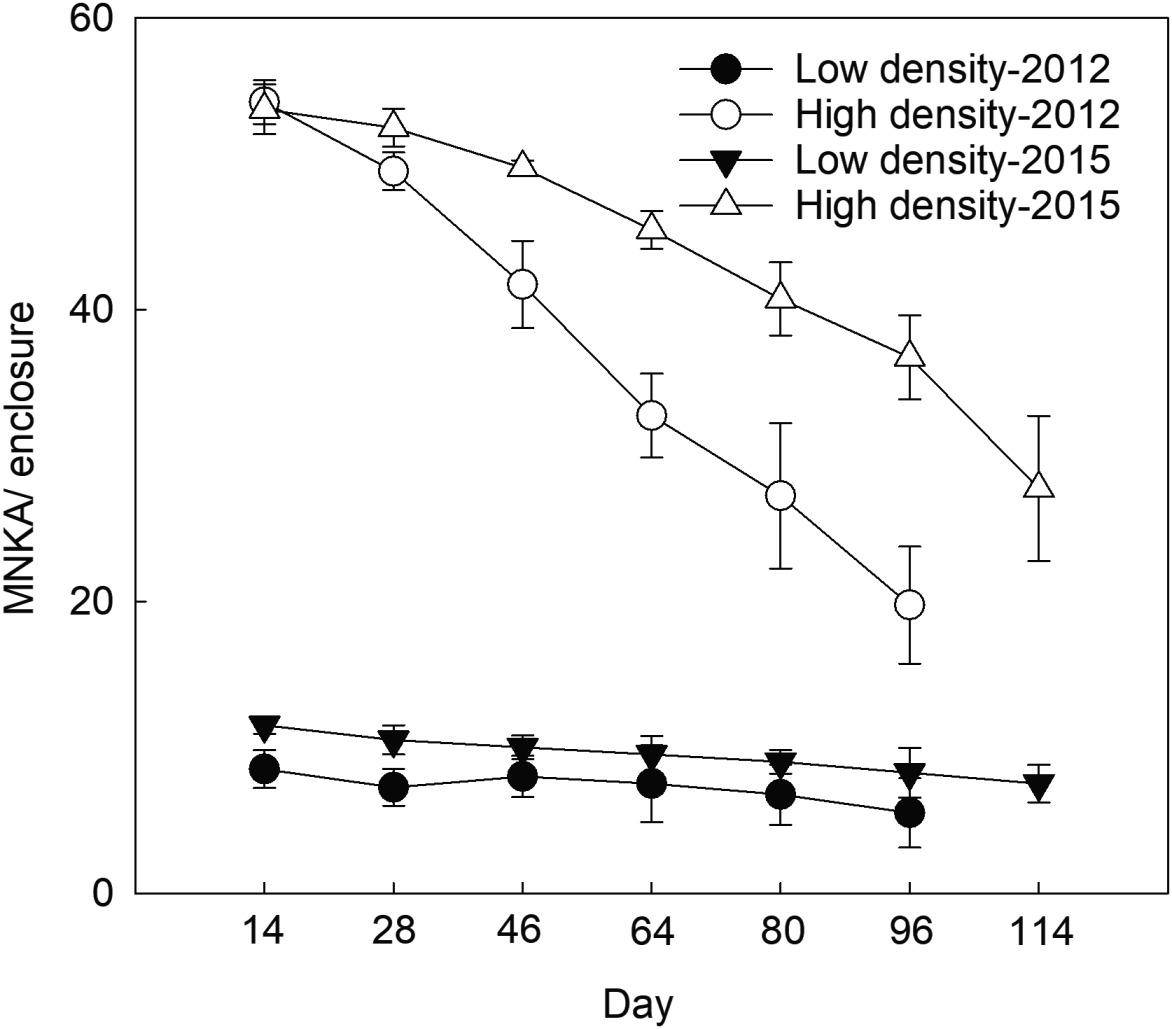
Founder numbers across live‐trapping sessions in 2012 and 2015. The population density was estimated as the minimum number known to be alive (MNKA) in each enclosure. Data from the four enclosures in each of the two density groups were expressed as mean ± standard error (SE)

For the founder numbers, we found an effect of treatment, time, and interaction between treatment and time (Table 1). Where the number of founders was significantly higher in high‐density treatment than in low‐density treatment throughout the experiment, founder numbers decreased progressively following the trapping sessions in both years in the two treatments (Figure 1). There were no effect of sex and interactive effects of treatment, sex, and time except for the interaction between treatment and time (Table 1). The mean estimated numbers of founder in the low‐density treatment during the experiment were 3.63 ± 0.24 in 2012 and 4.73 ± 1.21 in 2015. The mean numbers in the high‐density treatments were18.77 ± 1.21 in 2012 and 21.59 ± 0.71 in 2015.

**TABLE 1:** Generalized linear mixed model (GLMM) analyses of founder numbers, recruitment rate, proportion of reproductive females and males, and recapture rates

	Factors	df 1	df 2	*F*	*p*
Founder numbers	Treatment	**1**	**180**	**134.26**	**<.001**
	Sex	1	180	2.06	.153
	Time	**6**	**180**	**11.85**	**<.001**
	Treatment × sex	1	180	0.00	.969
	Treatment × time	**6**	**180**	**2.26**	.**040**
	Sex × time	6	180	0.88	.511
	Treatment × sex × time	6	180	1.03	.408
Recruitment rate	Treatment	**1**	**62**	**18.34**	**<.001**
	Time	4	62	0.12	.975
	Treatment × time	4	62	0.62	.650
Proportion of reproductive females	Treatment	**1**	**90**	**13.44**	**<.001**
	Time	**6**	**90**	**2.47**	.**030**
	Treatment × time	6	90	1.04	.405
Proportion of reproductive males	Treatment	**1**	**90**	**14.05**	**<.001**
	Time	**6**	**90**	**10.20**	**<.001**
	Treatment × time	6	90	0.61	.720
Recapture rates	Treatment	**1**	**193**	**14.42**	**<.001**
	Time	**6**	**193**	**22.53**	**<.001**
	Treatment × time	**6**	**193**	**4.17**	.**001**

Note

The significant main effects and interactions are in bold.

**FIGURE 2:**
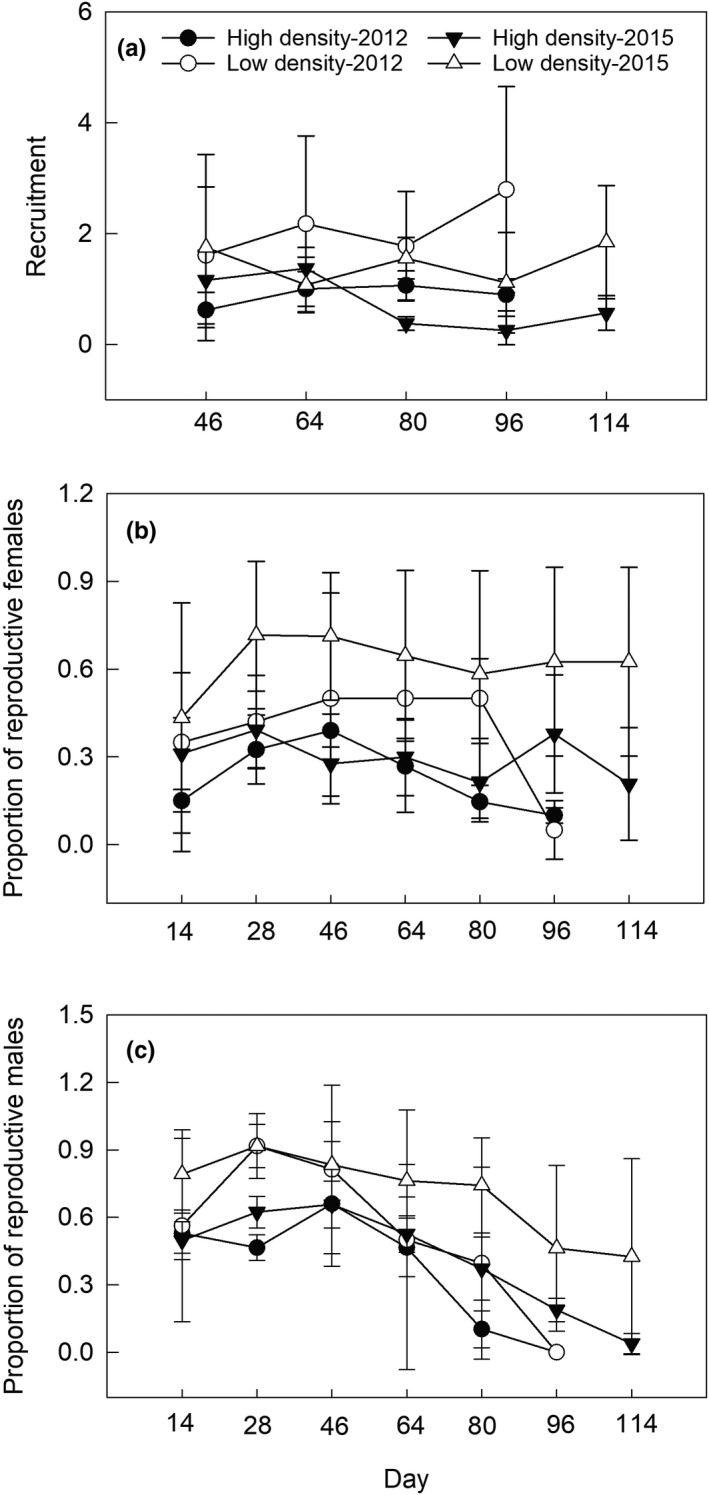
Mean (± SE) recruitment rate in 2012 and 2015 (a), mean proportion of reproductively active females in 2012 and 2015 (b), and of reproductively active males in 2012 and 2015 (c), in the experimental periods. Data from the four enclosures in each of the two density groups were expressed as mean ± standard error (SE)

For recruitment rate, we verified an effect of treatment and time, but no interaction between treatment and time (Table 1). Populations in low‐density enclosures in 2012 and 2015 had 131% and 97% higher recruitment rate than those in high‐density enclosures, respectively (Figure 2a).

For the proportion of reproductive females, we found an effect of treatment and time, but no effects of time or interaction between treatment and time (Table 1). For males, we found an effect of treatment and time, but interactions between treatment and time were not found (Table 1). Populations in low‐density enclosures in 2012 and 2015 had 70% and 107% higher proportions of reproductive females and 38% and 69% higher proportions of reproductive males than populations in high‐density enclosures, respectively (Figure 2b,c).

**FIGURE 3:**
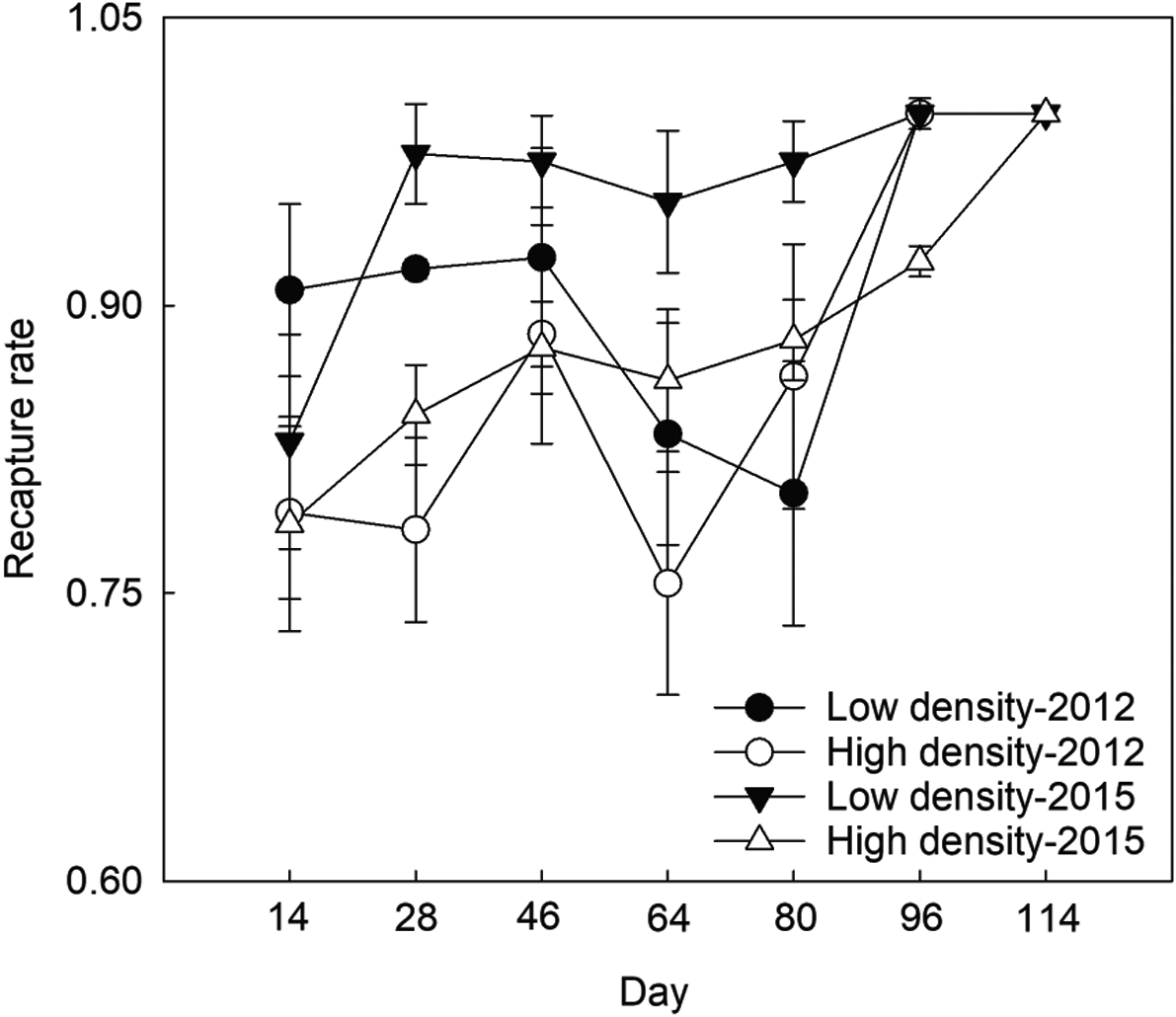
Recapture rate of the low‐ and high‐density population in 2012 and 2015, in the experimental periods. Data were expressed as mean ± standard error (SE)

In addition, there was significant difference in recapture rate between low and high density (Table 1), where recapture rate in low density was significantly higher than those in high density in the two trapping session of late June and August (Figure 3).

### 3.2 Direct and indirect effects of density on FCM level and reproductive traits

**FIGURE 4:**
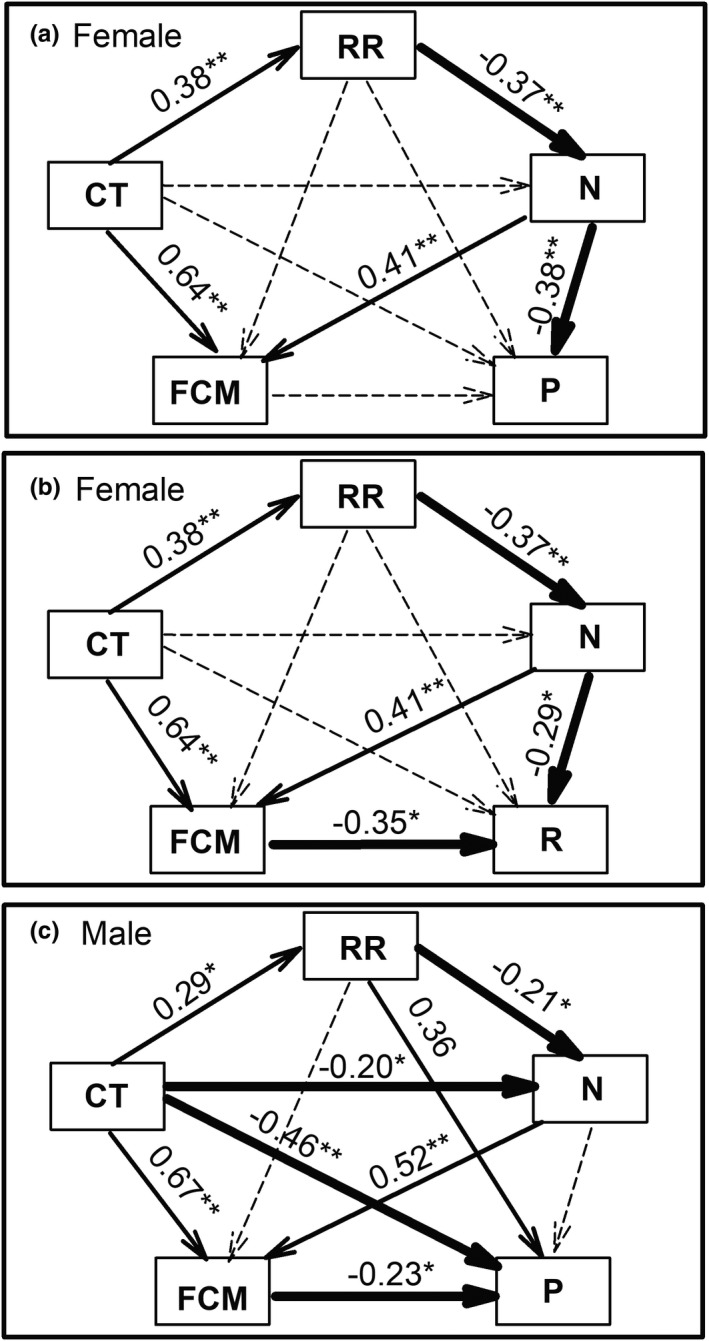
Recursive models in structural equation model, testing the direct and indirect effects of founder number and FCM level on proportion of reproductive females (a) and males (c), and on female recruitment rate (b). The structural equation model considered all plausible pathways in which trapping sessions, number, and FCM levels influence reproduction. Arrows represent the flow of causality. Thin solid represent a significant positive pathway; bold solid, a significant negative pathway; and dotted arrows, a non‐significant pathway. Path coefficients associated with each arrow show standardized effect sizes. Numbers indicate the standard path coefficients. CT, cumulative time of trapping session; RR, recapture rate; N, number of founders; P, proportion of reproductively active individuals; R, recruitment rate. * *p* < .05, **p < .001. For the proportion of reproductive females, *χ* ^2^ = 6.87, *p* = .23, RMSEA = .05. For the proportion of reproductive males, *χ* ^2^ = 2.19, *p* = .33, RMSEA = .03. For recruitment rate, *χ* ^2^ = 4.14, *p* = .39, RMSEA = .02. RMSEA, root mean square error of approximation

SEM showed that the number of female or male founders had positive association with FCM level and there was no association between recapture rate and FCM level (Figure 4). The number of female founder directly affected the proportion of reproductive females (Figure 4a) and recruitment rate (Figure 4b), in addition, the number of female founder indirectly affected recruitment rate through FCM level (Figure 4b). For males (Figure 4c), the effect of the number of male founders on the proportion of reproductive males was mediated by FCM level.

## 4 DISCUSSION

In the present study, high‐density treatment had higher founder numbers and lower recruitment rate and proportion of reproductively active individuals than low‐density treatment. Because offspring born in enclosures were moved to the laboratory to use to examine the effects of density‐induced maternal stress on offspring phenotype (Bian et al., 2015; Yang et al., 2018), the negative effect on reproductive traits in high‐density treatment was only due to the suppressive effects of founder numbers on reproduction. In addition, because the enclosures were isolated from common vole predators, predator‐induced density dependence on demographic processes was excluded from our study. Therefore, our results concluded that high density can induce the negative effect of density‐dependent reproduction, which corroborates other studies on vole populations as described in the Introduction section, indicating a universal of negative density‐dependent reproduction in the population of microtine rodents.

**FIGURE 5:**
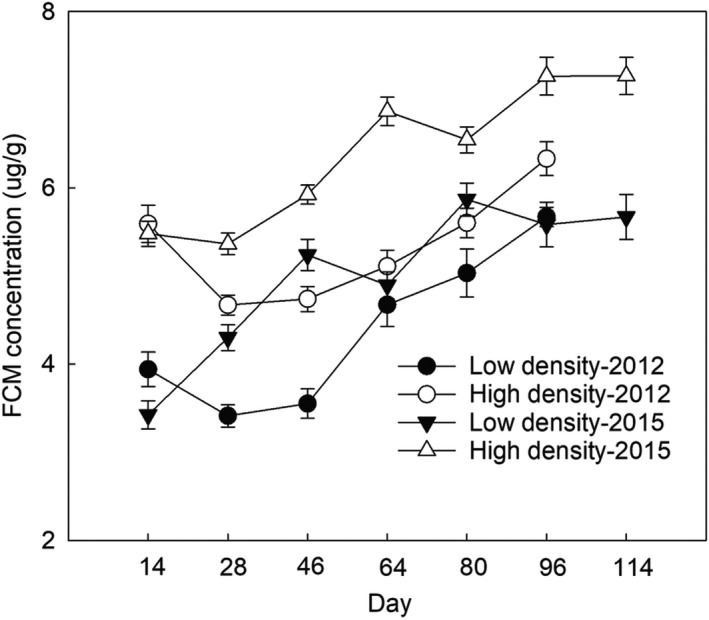
Fecal corticosterone metabolite (FCM) levels of founders in 2012 and 2015. Data have been published in our previous papers (Bian et al., 2015; Yang et al., 2018)

In our previous papers, we have reported that high‐density population in both years had higher FCM level than low‐density population (Bian et al., 2015; Yang et al., 2018; Figure 5). In the present study, we also revealed that FCM levels were positively associated with founder numbers, which also corroborates recent studies on other mammalian species (Boonstra & Boag, 1992; Novikov & Moshkin, 1998; Viblanc et al., 2014). However, Charbonnel et al. (2008) and Harper and Austad (2004) did not find a positive correlation between density and FCM levels in water voles and red‐backed voles (*Clethrionomys gapperi*). In those studies, the feces were sampled in both breeding and non‐breeding season (Charbonnel et al., 2008; Harper & Austad, 2004) and some samples were from individuals of different ages and reproduction conditions (Harper & Austad, 2004). However, our experiment was performed during the breeding season and the fecal samples of pregnant individuals were not collected due to pregnancy naturally raising glucocorticoid levels in most mammals, not necessarily as a result of stress but for developmental and energetic reasons (Boonstra & Boag, 1992; Edwards & Boonstra, 2018; Edwards et al., 2019). Our previous study has validated the effectiveness of detecting corticosterone levels in the feces of root voles (He et al., 2013). In addition, recapture rate may be a factor to affect stress responses. Although the difference in the recapture rate existed in our experiment, low‐density treatment had higher recapture rate than high‐density treatment, but it is no association with FCM levels, indicating that recapture rate was not a factor to be responsible for the high FCM level in high‐density treatment. Therefore, our experiment excluded the confounding effects of reproductively active individuals, seasonality, and the effects of trapping/handling stress on FCM levels, and the difference between ours and those results may be due to these confounding factors.

In addition, for unknown reasons, when founder voles were introduced into enclosures to establish an enclosed population, a few voles died during the 2‐week period of acclimation to their new environments. At the first trapping session in 2012 and 2015, the mean number of founders in high‐density treatments were 54.5 and 53.8 voles per enclosure (363 and 358 vole ha^−1^), respectively. In this study area, Sun et al. (2002) reported that population density of root vole was 356 vole ha^−1^ in October in the habitat with mainly *E*. *nutan*s, where grazing activities were limited. Moreover, Rodd and Boonstra (1984) reported that the high density of meadow voles (*M*. *pennsylvanicus*) population in abandoned farmland reached 469 vole ha^−1^ in October. Although we realize that the high‐density treatments (400 vole ha^−1^) were the approximate peak density of natural vole populations in this study area, it is not an unseen natural extreme level. Thus, our findings of correlation between FCM levels and density can represent what happens in a natural vole population.

In semelparous and partially semelparous species (i.e., Australian semelparous marsupial, the arctic ground squirrel *Spermophilus parryii plesius*) and dominant individuals in social species and cooperative breeders (i.e., the African wild dog *Lycaon pictus*, dwarf mongoose *Helogale parvula*, and grey wolf *Canis lupus*), dominance was strongly correlated with reproductive success. However, dominance was also associated with heightened glucocorticoid levels, which reflected either a classical trade‐off of reproductive success for potential survival during short mating periods (2–3 weeks), only one breeding period followed by programmed death (see review by Boonstra, 2005), or an adaptive stress response for competing intensely for access to females and maintaining dominant status by frequent physical aggression and challenges (see review by Sapolsky, 2005). Conversely, iteroparous voles breed continuously throughout the breeding season, and antagonistic interaction is not particularly severe. For example, breeding male meadow voles do not appear to be engaging in costly aggressive acts to assert dominance or access females (Edwards et al., 2019). Dominant status is maintained by cues rather than physical combat; subordinates tend to have the highest indices of stress. Thus, for these iteroparous species, elevated corticosterone induced by high population density can suppress reproduction (Boonstra, 2005; Wingfield & Sapolsky, 2003). In fact, antagonistic behavior is not the only factor that induces stress responses; increased population or breeding density can also lead to an increase in parasite load, attraction of predators, and food shortage. These factors can induce stress responses in individuals (Creel et al., 2013). Thus, the density‐induced stress responses are an additive stress effect of intrinsic and extrinsic factors, reflecting the biological cost of cumulative stress responses (Goymann & Wingfield, 2004) rather than a single factor effect (i.e., antagonistic behavior).

In the present study, we found that female‐founder vole numbers directly affected the proportion of reproductive females, but the influence of male‐founder vole numbers on the proportion of reproductive males was mediated through FCM levels, and that female‐founder number directly and indirectly through FCM levels negatively affected recruitment rate. Although the influence of FCM levels on reproductive traits had different pathways for female and male, we still conclude that density‐induced stress participates in density‐dependent negative regulation of reproduction; also, density‐induced stress is one of the factors generating negative effects of density‐dependent reproduction.

In conclusion, in the present study, high density increased FCM levels of founders and decreased reproduction. The negative effect of high density on reproduction was partly through its positive effects on FCM levels of founder voles. Thus, our results provide the first evidence that density‐induced stress is one ecological factor generating negative density‐dependent reproduction in vole populations.

## AUTHOR CONTRIBUTIONS

**Guozhen Shang:** Data curation (equal); Writing – original draft (equal); Writing – review & editing (equal). **Shouyang Du:** Investigation (equal). **Yanbin Yang:** Investigation (equal). **Yan Wu:** Funding acquisition (equal); Writing – review & editing (equal). **Yifan Cao:** Investigation (equal). **Jianghui Bian:** Data curation (equal); Funding acquisition (equal); Project administration (equal); Writing – original draft (equal); Writing – review & editing (equal).

## CONFLICT OF INTEREST

The authors declare that they have no conflicts of interest.

## Data Availability

Study data are publicly available in Dryad Digital Repository (https://doi.org/10.5061/dryad.2280gb5sj).

## References

[ece38927-bib-0001] Arcese, P. , & Smith, J. N. (1988). Effects of population density and supplemental food on reproduction in song sparrows. Journal of Animal Ecology, 57(1), 119–136. 10.2307/4768

[ece38927-bib-0002] Bian, J. H. , Du, S. Y. , Wu, Y. , Cao, Y. F. , Nie, X. H. , He, H. , & You, Z. B. (2015). Maternal effects and population regulation: maternal density‐induced reproduction suppression impairs offspring capacity in response to immediate environment in root voles *Microtus oeconomus* . Journal of Animal Ecology, 84(2), 326–336. 10.1111/1365-2656.12307 25327547

[ece38927-bib-0003] Bian, J. H. , Fan, N. C. , Zeng, J. C. , & Shi, Y. Z. (1994). Studies on the successive relation between small mammal community and plant community in Alpine meadow. Acta Theriologica Sinica, 14(3), 209–216 (in Chinese, English abstract). 10.16829/j.slxb.1994.03.009

[ece38927-bib-0004] Boonstra, R. (2005). Equipped for life: the adaptive role of the stress axis in male mammals. Journal of Mammalogy, 86(2), 236–247. 10.1644/BHE-001.1

[ece38927-bib-0005] Boonstra, R. , & Boag, P. T. (1992). Spring declines in *Microtus pennsylvanicus* and the role of steroid hormones. Journal of Animal Ecology, 61(2), 339–352. 10.2307/5326

[ece38927-bib-0006] Both, C. (1998). Density dependence of clutch size: habitat heterogeneity or individual adjustment? Journal of Animal Ecology, 67(4), 659–666. 10.1046/j.1365-2656.1998.00227.x

[ece38927-bib-0007] Charbonnel, N. , Chaval, Y. , Berthier, K. , Deter, J. , Morand, S. , Palme, R. , & Cosson, J. F. (2008). Stress and demographic decline: a potential effect mediated by impairment of reproduction and immune function in cyclic vole populations. Physiological and Biochemical Zoology, 81(1), 63–73. 10.1086/523306 18040973

[ece38927-bib-0008] Chen, F. , Du, S. Y. , Bian, J. H. , You, Z. B. , & Wu, Y. (2012). Chronic hypoxia exposure during pregnancy is associated with a decreased active nursing activity in mother and an abnormal birth weight and postnatal growth in offspring of rats. Hormones and Behavior, 61(4), 504–511. 10.1016/j.yhbeh.2012.01.009 22285933

[ece38927-bib-0009] Christian, J. J. (1971). Population density and reproductive efficiency. Biology of Reproduction, 4(3), 248–294. 10.1093/biolreprod/4.3.248 4329718

[ece38927-bib-0010] Coulson, T. , Milner‐Gulland, E. J. , & Clutton‐Brock, T. (2000). The relative roles of density and climatic variation on population dynamics and fecundity rates in three contrasting ungulate species. Proceedings of the Royal Society of London. Series B: Biological Sciences, 267(1454), 1771–1779. 10.1098/rspb.2000.1209 PMC1690729 12233776

[ece38927-bib-0011] Creel, S. , Dantzer, B. , Goymann, W. , & Rubenstein, D. R. (2013). The ecology of stress: effects of the social environment. Functional Ecology, 27(1), 66–80. 10.1111/j.1365-2435.2012.02029.x

[ece38927-bib-0012] Dhondt, A. A. , Kempenaers, B. , & Adriaensen, F. (1992). Density‐dependent clutch size caused by habitat heterogeneity. Journal of Animal Ecology, 61(3), 643–648. 10.2307/5619

[ece38927-bib-0013] Edeline, E. , Haugen, T. O. , Weltzien, F. A. , Claessen, D. , Winfield, I. J. , Stenseth, N. C. , & Vøllestad, L. A. (2010). Body downsizing caused by non‐consumptive social stress severely depresses population growth rate. Proceedings of the Royal Society B: Biological Sciences, 277(1683), 843–851. 10.1098/rspb.2009.1724 PMC2842726 19923130

[ece38927-bib-0014] Edwards, P. D. , & Boonstra, R. (2018). Glucocorticoids and CBG during pregnancy in mammals: diversity, pattern, and function. General and Comparative Endocrinology, 259, 122–130. 10.1016/j.ygcen.2017.11.012 29155262

[ece38927-bib-0015] Edwards, P. D. , Dean, E. K. , Palme, R. , & Boonstra, R. (2019). Assessing space use in meadow voles: the relationship to reproduction and the stress axis. Journal of Mammalogy, 100(1), 4–12. 10.1093/jmammal/gyy161

[ece38927-bib-0016] Focardi, S. , Pelliccioni, E. , Petrucco, R. , & Toso, S. (2002). Spatial patterns and density dependence in the dynamics of a roe deer (*Capreolus capreolus*) population in central Italy. Oecologia, 130(3), 411–419. 10.1007/s00442-001-0825-0 28547048

[ece38927-bib-0017] Goymann, W. , & Wingfield, J. C. (2004). Allostatic load, social status and stress hormones: the costs of social status matter. Animal Behaviour, 67(3), 591–602. 10.1016/j.anbehav.2003.08.007

[ece38927-bib-0018] Harper, J. M. , & Austad, S. N. (2004). Fecal corticosteroid levels in free‐living populations of deer mice (*Peromyscus maniculatus*) and southern red‐backed voles (*Clethrionomys gapperi*). American Midland Naturalist, 152(2), 400–410.

[ece38927-bib-0019] He, H. , Cao, Y. F. , Chen, L. L. , Du, S. Y. , Nie, X. H. , & Bian, J. H. (2013). The utility of detecting corticosterone levels in feces of root vole (*Microtus oeconomus*). Acta Theirologica Sinica, 33(2), 164–171 (in Chinese, English abstract). 10.16829/j.slxb.2013.02.009

[ece38927-bib-0020] Inchausti, P. , Carslake, D. , Attié, C. , & Bretagnolle, V. (2009). Is there direct and delayed density dependent variation in population structure in a temperate European cyclic vole population? Oikos, 118(8), 1201–1211. 10.1111/j.1600-0706.2009.17488.x

[ece38927-bib-0021] Jiang, Y. J. , Wei, S. W. , Wang, Z. W. , Zhen, Y. W. , Cui, R. X. , & Sun, R. Y. (1991). Productivity investigation of the root vole (*Microtus oeconomus*) population in the Haibei alpine bushland (*Potentilia fruticosa*) I. Population dynamics. Acta Theirologica Sinica, 11(4), 270–278. (in Chinese, English abstract). 10.16829/j.slxb.1991.04.006

[ece38927-bib-0022] Koskela, E. , Mappes, T. , & Ylönen, H. (1999). Experimental manipulation of breeding density and litter size: effects on reproductive success in the bank vole. Journal of Animal Ecology, 68(3), 513–521. 10.1046/j.1365-2656.1999.00308.x

[ece38927-bib-0023] Krebs, C. J. , & Myers, J. H. (1974). Population cycles in small mammals. Advances in Ecological Research, 8, 267–399. 10.1016/S0065-2504(08)60280-9

[ece38927-bib-0024] Kuznetsov, V. A. , Tchabovsky, A. V. , Kolosova, I. E. , & Moshkin, M. P. (2004). Effect of habitat type and population density on the stress level of Midday Gerbils (*Meriones meridianus*) in free‐living population. Biology Bulletin of the Russian Academy of Sciences, 31(6), 628–632. 10.1023/B:BIBU.0000049736.02912.e2 15615454

[ece38927-bib-0025] Lee, A. K. , & McDonald, I. R. (1985). Stress and population regulation in small mammals. Oxford Reviews of Reproductive Biology, 7, 261–304. 3001617

[ece38927-bib-0026] Liu, J. K. , Wang, X. , & Liu, W. (1991). Studies on the nutritional ecology of small herbivorous mammals: Patterns of food selection and resource utilization for root voles and Gansu pikas. In J. Liu & Z. Wang (Eds.), Alpine meadow Ecosystem, Fasc 3 (pp. 111–124). Science Press.

[ece38927-bib-0027] Mcdonald, A. J. (1998). Cortical pathways to the mammalian amygdala. Progress in Neurobiology, 55(3), 257–332. 10.1016/S0301-0082(98)00003-3 9643556

[ece38927-bib-0028] Møller, A. P. (1989). Population dynamics of a declining swallow *Hirundo rustica* population. Journal of Animal Ecology, 58(3), 1051–1063. 10.2307/5141

[ece38927-bib-0029] Mugabo, M. , Galliard, J. F. L. , Perret, S. , Decencière, B. , Haussy, C. , & Meylan, S. (2017). Sex‐specific density‐dependent secretion of glucocorticoids in lizards: insights from laboratory and field experiments. Oikos, 126(7), 1051–1061. 10.1111/oik.03701

[ece38927-bib-0030] Novikov, E. , & Moshkin, M. (1998). Sexual maturation, adrenocortical function and population density of red‐backed vole, *Clethrionomysrutilus* . Mammalia, 62(4), 529–540. 10.1515/mamm.1998.62.4.529

[ece38927-bib-0031] Ostfeld, R. S. , Canham, C. D. , & Pugh, S. R. (1993). Intrinsic density‐dependent regulation of vole populations. Nature, 366(6452), 259–261. 10.1038/366259a0 8232583

[ece38927-bib-0032] Pihl, L. , & Hau, J. (2003). Faecal corticosterone and immunoglobulin A in young adult rats. Laboratory Animals, 37(2), 166–171. 10.1258/00236770360563822 12689429

[ece38927-bib-0033] Rodd, F. H. , & Boonstra, R. (1984). The spring decline in the meadow vole, *Microtus pennsylvanicus*: the effect of density. Canadian Journal of Zoology, 62(8), 1464–1473. 10.1139/z84-212

[ece38927-bib-0034] Rödel, H. G. , Bora, A. , Kaiser, J. , Kaetzke, P. , Khaschei, M. , & Von Holst, D. (2004). Density‐dependent reproduction in the European rabbit: a consequence of individual response and age‐dependent reproductive performance. Oikos, 104(3), 529–539. 10.1111/j.0030-1299.2004.12691.x

[ece38927-bib-0035] Saitoh, T. (1981). Control of female maturation in high density populations of the red‐backed vole, *Clethrionomys rufocanus bedfordiae* . Journal of Animal Ecology, 50(1), 79–87. 10.2307/4032

[ece38927-bib-0036] Saitoh, T. , Chr, N. , & Bjornstad, O. N. (1997). Density dependence in fluctuating grey‐sided vole populations. Journal of Animal Ecology, 66(1), 14–24. 10.2307/5960

[ece38927-bib-0037] Sapolsky, R. M. (2005). The influence of social hierarchy on Primate health. Science, 308(5722), 648–652. 10.1126/science.1106477 15860617

[ece38927-bib-0038] Saucy, F. (1994). Density Dependence in Time Series of the Fossorial Form of the Water Vole, *Arvicolaterrestris* . Oikos, 71(3), 381–392. 10.2307/3545826

[ece38927-bib-0039] Schreiber, J. B. (2008). Core reporting practices in structural equation modeling. Research in Social and Administrative Pharmacy, 4(2), 83–97. 10.1016/j.sapharm.2007.04.003 18555963

[ece38927-bib-0040] Sheriff, M. J. , Krebs, C. J. , & Boonstra, R. (2009). The sensitive hare: sublethal effects of predator stress on reproduction in snowshoe hares. Journal of Animal Ecology, 78(6), 1249–1258. 10.1111/j.1365-2656.2009.01552.x 19426257

[ece38927-bib-0041] Sheriff, M. J. , Krebs, C. J. , & Boonstra, R. (2010). Assessing stress in animal populations: Do fecal and plasma glucocorticoids tell the same story? General and Comparative Endocrinology, 166(3), 614–619. 10.1016/j.ygcen.2009.12.017 20051245

[ece38927-bib-0042] Sun, P. , Zhao, X. Q. , Xu, S. X. , Zhao, T. B. , & Zhao, W. (2002). Changes after snow of the population characteristic of root vole (*Microtus oeconomus*) in Haibei Alpine meadow. Acta Theriologica Sinica, 22(4), 318–320 (in Chinese, English abstract). 10.16829/j.slxb.2002.04.014

[ece38927-bib-0043] Sun, R. Y. , Zheng, S. W. , & Cui, R. X. (1982). Home range of the root vole *Microtus oeconomus* . Acta Theriologica Sinica, 2(2), 219–231 (in Chinese, English abstract). 10.16829/j.slxb.1982.02.014

[ece38927-bib-0044] Viblanc, V. A. , Gineste, B. , Stier, A. , Robin, J. P. , & Groscolas, R. (2014). Stress hormones in relation to breeding status and territory location in colonial king penguin: a role for social density? Oecologia, 175(3), 763–772. 10.1007/s00442-014-2942-6 24744279

[ece38927-bib-0045] Wauters, L. A. , & Lens, A. (1995). Effects of food availability and density on red squirrel (*Sciurus vulgaris*) reproduction. Ecology, 76(8), 2460–2469. 10.2307/2265820

[ece38927-bib-0046] Wingfield, J. C. , & Sapolsky, R. M. (2003). Reproduction and resistance to stress: when and how. Journal of Neuroendocrinology, 15(8), 711–724. 10.1046/j.1365-2826.2003.01033.x 12834431

[ece38927-bib-0047] Yang, Y. B. , Shang, G. Z. , Du, S. Y. , Zhang, X. , Wu, Y. , & Bian, J. H. (2018). Maternal density stress and coccidian parasitism: Synergistic effects on overwinter survival in root voles. Functional Ecology, 32(9), 2181–2193. 10.1111/1365-2435.13129

[ece38927-bib-0048] Zhang, Z. B. , Xu, L. , Guo, C. , Wang, Y. , & Guo, Y. W. (2010). Effect of ENSO‐driven precipitation on population irruptions of the Yangtze vole *Microtus fortis calamorum* in the Dongting Lake region of China. Integrative Zoology, 5(2), 176–184. 10.1111/j.1749-4877.2010.00199.x 21392335

